# Characterization of Atherosclerosis Formation in a Murine Model of Type IIa Human Familial Hypercholesterolemia

**DOI:** 10.1155/2018/1878964

**Published:** 2018-06-07

**Authors:** Chiharu Miyajima, Takayuki Iwaki, Kazuo Umemura, Victoria A. Ploplis, Francis J. Castellino

**Affiliations:** ^1^Department of Pharmacology, Hamamatsu University School of Medicine, Hamamatsu 431-3192, Japan; ^2^The W. M. Keck Center for Transgene Research and the Department of Chemistry and Biochemistry, University of Notre Dame, Notre Dame, IN 46556, USA

## Abstract

A murine genetic model of LDL-cholesterol- (LDL-C-) driven atherosclerosis, based on complete deficiencies of both the LDL-receptor (*Ldlr*^−/−^) and key catalytic component of an apolipoprotein B-edisome complex (*Apobec1*^−/−^), which converts apoB-100 to apoB-48, has been extensively characterized. These gene deficiencies allow high levels of apoB-100 to be present and inefficiently cleared, thus leading to very high levels of LDL-C in mice on a normal diet. Many key features of atherosclerotic plaques observed in human familial hypercholesterolemia are found in these mice as they are allowed to age through 72 weeks. The general characteristics include the presence of high levels of LDL-C in plasma and macrophage-related fatty streak formation in the aortic tree, which progressively worsens with age. More specifically, plaque found in the aortic sinuses contains a lipid core with relatively high numbers of macrophages and a smooth muscle cell *α*-actin- and collagen-containing cap, which thins with age. These critical features of plaque progression suggest that the* Ldlr*^−/−^/*Apobec1*^−/−^ mouse line presents a superior model of LDL-C-driven atherosclerosis.

## 1. Introduction

Atherosclerosis is a self-sustaining inflammatory fibroproliferative disease that progresses in discrete stages and involves a number of cell types and effector molecules [1, 2]. Lipid metabolic disorders, principally those dyslipidemias that lead to high LDL and triglyceride levels and/or low HDL levels, are heavily involved in the genesis and progression of atherosclerosis in humans, and thrombotic complications substantially contribute to the coronary end-stage arterial disease that sometimes accompanies atherosclerosis. Because of genetic differences in lipid metabolism between mice and humans, LDL and its precursors, VLDLs and IDLs, are rapidly cleared in mice. Thus, differing from humans, the cholesterol present in murine plasma is mostly carried in the artheroprotective HDL fraction. As a result, wild-type (WT) mice are more resistant than humans to dietary-induced elevation of LDL and, thus, useful metabolic murine models of human atherosclerosis were not prevalent until recent advances in in vivo gene targeting methods. Application of this technology then allowed generation of genetic strains of mice that possess some characteristics of lipid metabolism of humans.

While accelerated injury- and transplant-based atherosclerosis, as well as spontaneous genetic models of Type II familial hypercholesterolemia (FH), the most frequent type of FH observed in humans that leads to atherosclerosis, are continually emerging, the most widely studied murine models of FH-mediated human atherosclerosis are the low density lipoprotein receptor-deficient (*Ldlr*^−/−^) [3] and apoE deficient (*Apoe*^−/−^) [4–6] mouse lines. In the former case, by allowing LDL elevation in plasma via elimination of the apoB-100 receptors,* Ldlr*^−/−^ mice provide a potential model of atherosclerosis elevated LDL-containing cholesterol (LDL-C) levels. However, differing from the human disease, these mice, when placed on a normal chow diet, present with only 2-fold elevated cholesterol concentrations and develop atherosclerosis very slowly [3]. High-fat, high-cholesterol diets are required for development of aortic atherosclerotic lesions in these mice [7] although VLDL-C not LDL-C is extremely elevated in these diets. With regard to the* Apoe*^−/−^ mouse strain, these mice display very high levels of plasma cholesterol and severe atherosclerosis, which is enhanced by a high-cholesterol diet [5]. However, the cholesterol is mainly associated with the VLDL and IDL lipoprotein fractions, not the LDL fraction, as in the case of humans. Thus, while nonetheless very valuable, neither of these models closely reflects the lipid profiles of the disease in humans. This is, in part, due to the presence of an enzyme in mouse liver, apobec-1, a RNA-specific cytidine deaminase. This enzyme is a catalytic component of an edisome that alters the apoB-100-encoding mRNA to an mRNA coding for apoB-48. Incorporation of apoB-48 into VLDL initiates its rapid clearance by scavenger receptors prior to conversion of VLDL into LDL particles. This editing, while also occurring in human intestines, does not take place in human liver, and its occurrence in mouse liver results in resistance to elevation in LDL in mice.

VLDL-C driven atherosclerotic plaques in* Apoe*^−/−^ and* Ldlr*^−/−^ mice are lipid-rich and abundant with the presence of foam cells converted from macrophages [8] and contain thinner fibrous cap and extremely small extracellular matrices [9]. In contrast, mainly LDL-C driven human arteriosclerotic plaques are extracellular matrix-rich and abundant with the presence of smooth muscle cells, and they are erosion-prone and vulnerable [10]. It is not known whether such differences are due to species or cholesterol profile.

Because of the limited utility of the* Ldlr*^^−/−^^ and* Apoe*^−/−^ mouse lines in terms of lipid metabolism, other murine models have been developed that have focused on elevation of apoB-100 levels in mice. Among these models are the apoB-100 transgenic strain (*(Tg)APOB100*^+/+^) [11] and mice with a combined* (Tg)APOB100*^+/+^/*Ldlr*^−/−^ genotype [12]. While this latter model possesses important advantages in lipid metabolism, it should be emphasized that introducing other gene modifications to this line is difficult because the* APOB100* loci are not fixed. A potentially important mouse model of human atherosclerosis with elevated LDL-C is one in which both the* Ldlr* and* Apobec1* genes are deleted (*Ldlr*^−/−^/*Apobec1*^−/−^, hereafter referred to as* L*^−/−^*/A*^−/−^), since these mice lack the ability to convert apoB-100 to apoB-48 in liver and also are defective in LDL clearance. These doubly deficient mice exhibit high levels of apoB-100-LDL-C, more closely mirroring the plasma lipid profiles in human Type II FH [13], and slowly and progressively present with severe spontaneous atherosclerosis on a normal chow diet. Thus, a good model is potentially available to further test the evolution of the atheroma and its* in vivo* relationships with specific proteins. While some initial characterizations of this murine model have been published, a thorough analysis of plaque development and progression is warranted. The current manuscript presents results of an investigation in which the nature of the plaque has been characterized over the majority of the lifespan of the* L*^−/−^/*A*^−/−^ mouse.

## 2. Materials And Methods

### 2.1. Mice

*L* ^−/−^/*A*^−/−^ mice have been initially described [13, 14]. These mice were back-crossed to C57Bl6/J mice (Jackson Laboratory, Bar Harbor, ME) for at least seven generations before cross-breeding. Each genotype was determined using PCR analysis with genomic DNA from ear punch biopsy. Male mice were used in all experiments. The mice were maintained on a low-fat diet for 12, 18, 24, 36, 48, 60, and 72 weeks. Mice fasted at least 6 h and sacrificed at each time point using overinhalation of isoflurane, and then their blood was obtained with sodium citrate or heparin as an anticoagulant. Their hearts and whole aortic trees were removed for morphometric analyses after perfusion with isotonic saline. All animal experiments described herein were approved by the Institutional Animal Care and Use Committee (IACUC) at the University of Notre Dame and Hamamatsu University School of Medicine.

### 2.2. Lipid Analysis of Whole Plasma

Plasma was separated from whole citrated blood and used for the measurement of total cholesterol and triglycerides employing the cholesterol CII (Wako Chemicals, Richmond, VA) and GPO TRINDER kits (Sigma Diagnostics, St. Louis, MO), respectively. For the assay, a volume of 2 *μ*L of plasma was mixed with 100 *μ*L of the assay reagent from each kit and incubated at 37°C for 30 min. The absorbancies at 500 nm and 540 nm were obtained for total cholesterol and triglyceride, respectively, and calculated using the standards present in each kit. These procedures were as described in our previous report [15].

### 2.3. FPLC Analysis of Whole Plasma

A volume of 100 *μ*L of plasma was analyzed by FPLC, using gel filtration on Superose 6 HR resin (Amersham Pharmacia Biotech, Piscataway, NJ). The samples were eluted at a flow rate of 0.5 mL/min with a column equilibration buffer, namely, 10 mM Tris-HCl/0.15 M NaCl/0.01% (w/v) EDTA, pH 7.4, as previously described [6]. Column volumes (500 *μ*L) were collected (36 fractions) and a 50 *μ*L aliquot from each tube was added to 100 *μ*L of cholesterol CII reagent for the determination of the cholesterol concentration in each fraction. These procedures were as described in our previous report [15].

### 2.4. Analysis of Atherosclerotic Lesions of Whole Aortic Trees

After perfusion of the mice, the appearance of the aortic arch was photographed, and then the aortas were exposed and cut longitudinally, in situ, exposing the lumen. Whole aortic trees were removed and placed on 150 *μ*m gapped glass slides, with the lumen-side up. A glass slide was then placed over the lumens of the aortas to hold them in place during fixation. The aortas were then fixed in 10% normal buffered formalin for 16 hr at room temperature. They were rinsed with H_2_O and stained with Sudan IV (Sigma) solution (38% 2-propanol with supersaturated Sudan IV) for 16 hr at 4°C. A digital camera was used to capture the whole image and the total number of pixels for whole aortas and plaque areas were measured using Adobe Photoshop 7.0, thus allowing calculation of the % of the total surface area of plaque in the aortas. These procedures were as described in our previous report [15].

### 2.5. Sections of Hearts

Hearts were cut at the level of the lower edge of the atrium and the lower region containing the aortic valve was fixed with periodate-lysine-paraformaldehyde (PLP) for 16 h at 4°C. After fixation, some samples were directly embedded in Tissue-Tec OCT compound (Sakura Fine Tec, Torrance, CA), and others were processed and embedded in paraffin. A total of 30 serial sections (from #1 to #30) were obtained at a 4 *μ*m thickness from the aortic valve towards the ascending aorta. These procedures were as described in our previous report [15].

### 2.6. Histochemistry for Analysis of Plaque Progression in Aortic Sinus

Serial sections (#1, #11, and #21) were stained with hematoxylin II and eosin Y (H&E) (Richard Allen Scientific, Kalamazoo, MI) for morphometric analysis. Serial sections (#2, #12, and #22) were stained with Oil Red-O (Sigma) for detecting lipid accumulation in the plaque. For the determination of the size of plaques in aortic sinuses, these images were captured and calculated as above, and the number of pixels was converted to *μ*m^2^ with a proper reference measurement (hemocytometer grid). Serial sections (#3, #13, and #23) were used for Masson's trichrome stain to identify collagen accumulation in the plaque. These procedures were as described in our previous report [15].

### 2.7. Immunohistochemistry

All slides were deparaffinized and then blocked with avidin block, biotin block, and Peroxo-block (Zymed Laboratories, South San Francisco, CA), before incubating in specific antibodies. For fibrin(ogen) staining, the slides were blocked only with Peroxo-block. These following procedures were as described in our previous report [15].

*Antifibrin(ogen) immunostaining. *Serial sections (#4, #14, and #24) were further blocked with normal rabbit serum. The sections were then incubated with a goat-anti-mouse fibrin(ogen) antibody (Nordic Immunology, Tilburg, The Netherlands), followed by rabbit anti-goat-IgG in 10% normal mouse serum. A complex of horseradish peroxidase (HRP), conjugated to a goat-anti-HRP-IgG solution (DAKO, Carpentaria, CA), was added. The slides were developed with 3-amino-9-ethylcarbazole (AEC), which was followed by a hematoxylin QS counterstain (Vector Labs, Burlingame, CA).

*Anti-CD31 Immunostaining for Platelet Endothelial Cell Adhesion Molecule 1 (PECAM1).* Serial sections (#5, #15, and #25) were further blocked with 10% normal rabbit serum and then incubated with a rat-anti-mouse-CD31 monoclonal antibody (Pharmingen, San Diego, CA), followed by biotinylated rabbit-anti-rat-IgG (DAKO) in 5% preimmune mouse serum. After adding streptavidin-HRP complex, the sections were developed with 3,3′-diaminobenzidine (DAB) followed by a hematoxylin QS counterstain.

*Anti-MAC3 and Anti-F4/80 Immunostaining for Macrophage Detection.* Serial sections (#6, #16, and #26) were further blocked with 10% normal goat serum and then incubated with a rat-anti-mouse-MAC3 monoclonal antibody (Pharmingen) and a rat-anti-mouse-F4/80 monoclonal antibody (Serotec, Raleigh, NC). This was followed by HRP-conjugated goat-anti rat-STAR-72 IgG (Serotec). SG chromogen (Vector Labs) was applied to the sections for positive staining and hematoxylin QS for counterstaining.

*Smooth Muscle Cells (SMCs). *Serial sections (#7, #17, and #27 and #8, #18, and #28) were incubated in 1 % sodium dodecyl sulfate/0.1 M phosphate-buffered saline (PBS), pH 7.3, for antigen retrieval, followed by the blocking steps described above. A non-serum protein block (DAKO) was additionally used to block nonspecific immunoglobulins. Sections #7, #17, and #27 were incubated with an SMC anti-*α*-actin (SMA) antibody (DAKO) or anti-myosin (nonmuscle) heavy chain (SMemb) antibody (Research Diagnostics, Flanders, NJ) (sections #8, #18, and #28), followed by HRP-conjugated goat-anti-rabbit-IgG. The slides were developed with AEC followed by a hematoxylin QS counterstain.

## 3. Results

**Figure 1:**
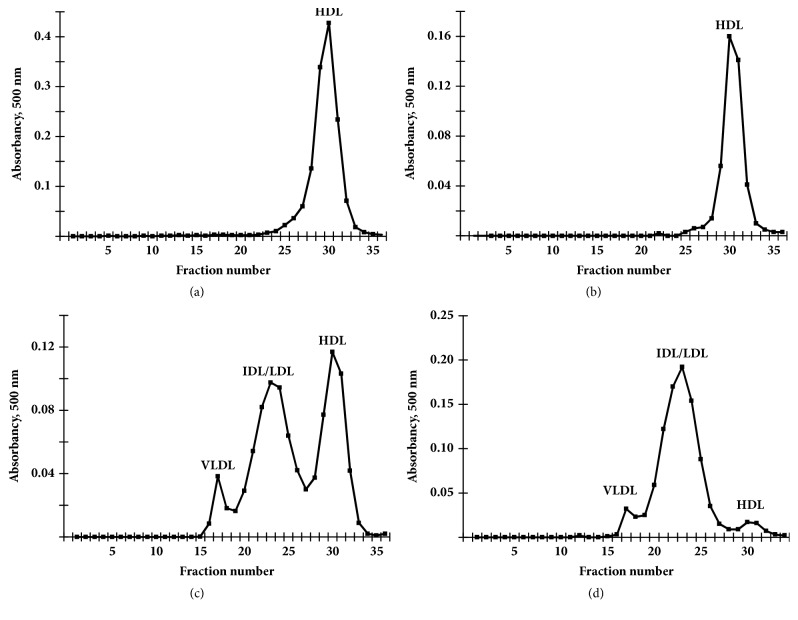
FPLC analysis, with cholesterol detection of (a) Wt, (b)* A*^−/−^ (c)* L*^−/−^, and (d)* L*^−/−^*/A*^−/−^ mouse plasma. In these profiles, VLDL fractions are 15-19, IDL/LDL fractions are 20-27, and HDL fractions are 28-32.

**Figure 2:**
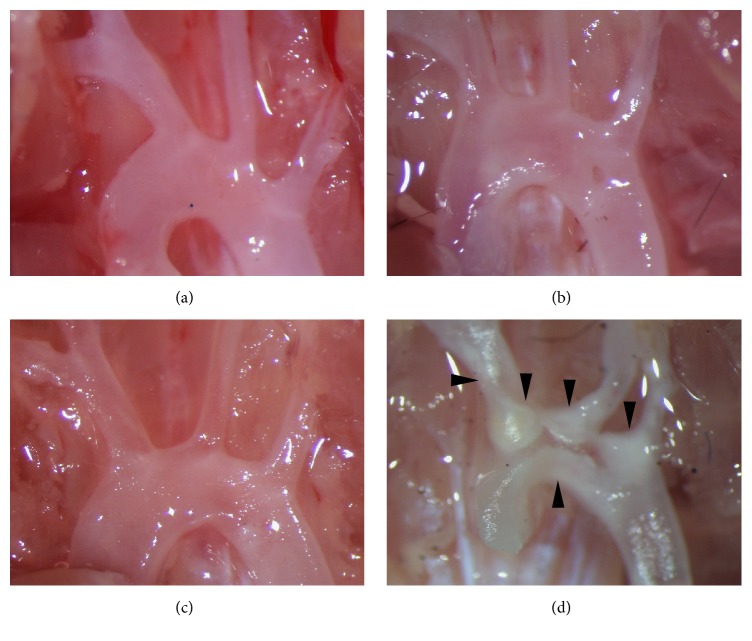
The appearance of aortic arch of (a) Wt, (b)* A*^−/−^ (c)* L*^−/−^, and (d)* L*^−/−^*/A*^−/−^ mice at 24 weeks of age. There are no clear atherosclerotic plaques in Wt (a) and* A*^−/−^ mice (b) and even in* L*^−/−^ mice (c) at this time point on normal chow diet. On the other hand, apparent atherosclerotic plaques are visible in branching portions and the aortic arch in* L*^−/−^*/A*^−/−^ mice (black arrowheads) (d).

**Figure 3:**
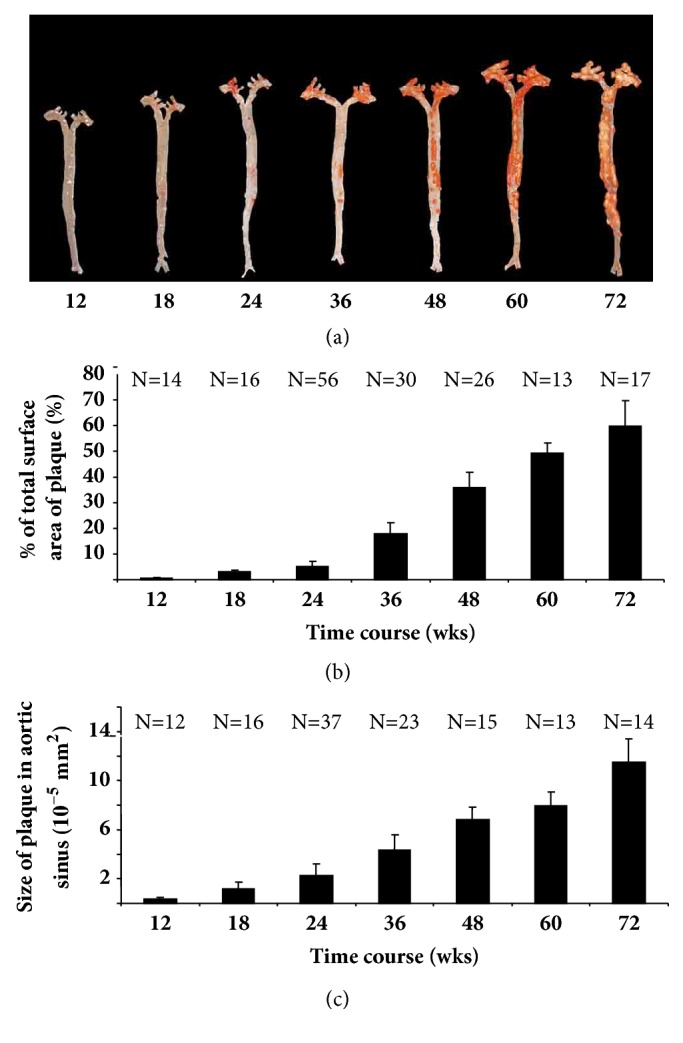
(a) Sudan IV staining of whole-mount aortic trees of* L*^−/−^*/A*^−/−^ mice at the indicated ages (weeks), showing the extent of lipid-containing plaque. (b) Percentage of total surface area of aortic trees that contains plaque as a function of age, in* L*^−/−^*/A*^−/−^ mice. (c) Plaque size in aortic sinuses of* L*^−/−^*/A*^−/−^ mice as revealed by morphometric analysis of H&E stained slides. Plaque sizes for each mouse are based on an average of 3 individual equally spaced sections of the aortic sinus. The pixel numbers are converted to *μ*m^2^ with use of a 1 mm x 1 mm calibrator. N = the number of mice used at each time point.

It is known that female mice are more vulnerable to atherosclerosis than male mice due to sex steroid hormone fluctuations [16, 17]. Thus, the progression of spontaneous atherosclerosis in only male mice maintained on a low-fat diet has been assessed. The distribution of cholesterol in the various lipoprotein fractions, as determined from FPLC profiles of the type shown in Figure 1, for mice aged up to 72 weeks, is listed in Table 1. The levels of these key lipids remain constant over this time in* L*^−/−^/*A*^−/−^ mice. Initially, Total-C and triglyceride levels in Wt,* A*^−/−^,* L*^−/−^, and* L*^−/−^*/A*^−/−^ mice at 24-weeks of age are compared. As expected, Total-C in Wt and* A*^−/−^ mice are very low, and most of them are packaged in the HDL fraction (Figures 1(a) and 1(b)). As opposed to* L*^−/−^ mice, where some are present in the LDL fraction (Figure 1(c)), and in* L*^−/−^/*A*^−/−^ mice, the bulk of the cholesterol is present in the LDL fraction (Figure 1(d)). As expected, no plaques are found in the aortic arch of Wt and* A*^−/−^ mice (Figures 2(a) and 2(b)). Although LDL-C is elevated in* L*^−/−^ mice, no obvious plaques are found in the aortic arch (Figure 2(c)) as previously reported [12]. In contrast, atherosclerotic plaques are present in the aortic arch of* L*^−/−^*/A*^−/−^ mice (Figure 2(d)). Thus,* L*^−/−^*/A*^−/−^ mice were used for further analyses. Sudan IV staining of the arterial trees (Figure 3(a)) shows that lipid-containing plaque forms initially in the aortic sinuses and then spreads throughout the entire aortic trees. The percentage of the trees containing plaque progresses from approximately 2% in mice 12 weeks of age to approximately 60% in 72 weeks (Figure 3(b)), thus showing the extensive nature of plaque deposition in these mice. Aortic sinuses have been microsectioned from* L*^−/−^/*A*^−/−^ mice. The sizes of the plaques in the aortic sinuses of* L*^−/−^/*A*^−/−^ mice were measured on 3 equally spaced H&E stained sections from each mouse aortic sinus. The data show that plaque continues to increase throughout the lifespans of the mice (Figure 3(c)). No significant plaque was found in Wt mice fed the same diet as young mice (12 weeks of age).

**Figure 4:**
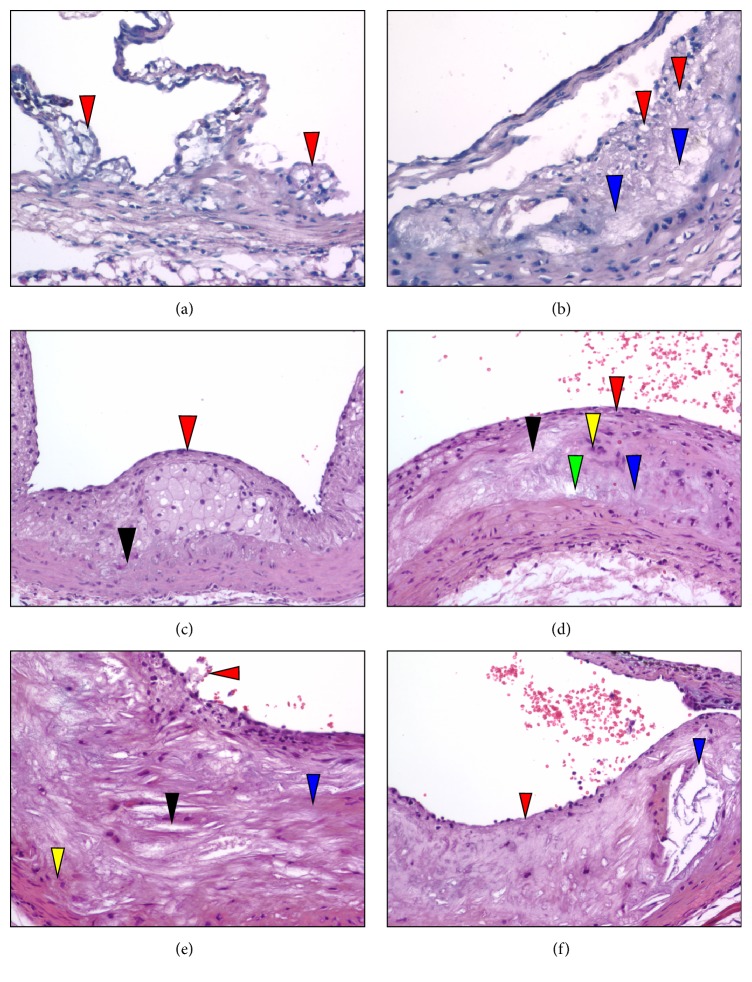
H&E stains of aortic sinuses from typical* L*^−/−^*/A*^−/−^ mouse at various ages. (a) 12 weeks of age. H&E staining reveals fatty deposits (red arrowheads) present in the small intimal compartment adjacent or attached to the base of the aortic valve. Original magnification, 200X. (b) 18 weeks of age. H&E stains display a diffuse thickening of the intimal compartment, consisting primarily of foam cells (red arrowheads) in addition to the appearance of focal acellular areas above the media (blue arrowheads). Original magnification, 200X. (c) 24 weeks of age. H&E stain demonstrating a well-formed fibrous cap (red arrowhead) encapsulating a foam cell laden core. Arterial wall distension (black arrowhead) is noted in the media associated with stretching of the elastic lamella. Original magnification, 100X. (d) 36 weeks of age. H&E stains indicate that, at this time point, an intermediate size lesion is evident. The cap consists of a thin layer of cells (red arrowhead) and focal acellular patches (blue arrowhead), cellular debris, extracellular lipids, and cholesterol clefts (black arrowhead) are observed within the core. Focal cellular aggregates within the core (yellow arrowhead) and arterial wall distention (green arrowhead) are evident. Original magnification, 100X. (e) 48 weeks of age. H&E stains show that at this later time point the thin cap consists of a monolayer to bilayer of cells with apparent breaks in the endothelium and sloughing of the cap (red arrowhead). Large acellular areas (blue arrowhead) are evident in the well-established lipid core and there are abundant cholesterol clefts (black arrowhead). The arterial wall appears stretched (yellow arrowhead). Original magnification, 100X. (f) 60 weeks of age. H&E staining shows that the complex lesion consists of a thin monolayer of cells in the cap region with intermittent acellular patches (red arrowhead). The acellular lipid core is expanded with calcium deposition and cholesterol clefts (blue arrowhead). Original magnification, 100X.

H&E stains of sectioned aortic sinuses from* L*^−/−^/*A*^−/−^ mice at various ages are presented in Figure 4. At 12 weeks of age, H&E staining reveals fatty deposits in the small intimal compartment adjacent or attached to the base of the aortic valve (Figure 4(a)). At 18 weeks of age, H&E staining shows a diffuse thickening of the neointima that is primarily due to foam cells and focal acellular areas above the media (Figure 4(b)). At 24 weeks of age, H&E staining demonstrates a well-formed fibrous cap overlaying a foam cell laden core. Arterial wall thickening, with collagen development and stretching of lamella (Figure 4(c)), is noted in the media. At 36 weeks of age, H&E stains (Figure 4(d)) indicate an intermediate size lesion. The cap consists of a thin layer of cells and focal acellular patches, while cellular debris and extracellular lipids are observed within the core. At 48 weeks of age, H&E stains (Figure 4(e)) show that at this later time point the thin cap consists of a monolayer to bilayer of cells with apparent breaks in the endothelium and sloughing of the cap. Most of the core is acellular, with abundant cholesterol clefts. At 60 weeks of age, H&E staining (Figure 4(f)) shows that the complex lesion consists of a thin monolayer of cells in the cap region with intermittent acellular patches. The acellular lipid core is expanded with cholesterol clefts.

**Figure 5:**
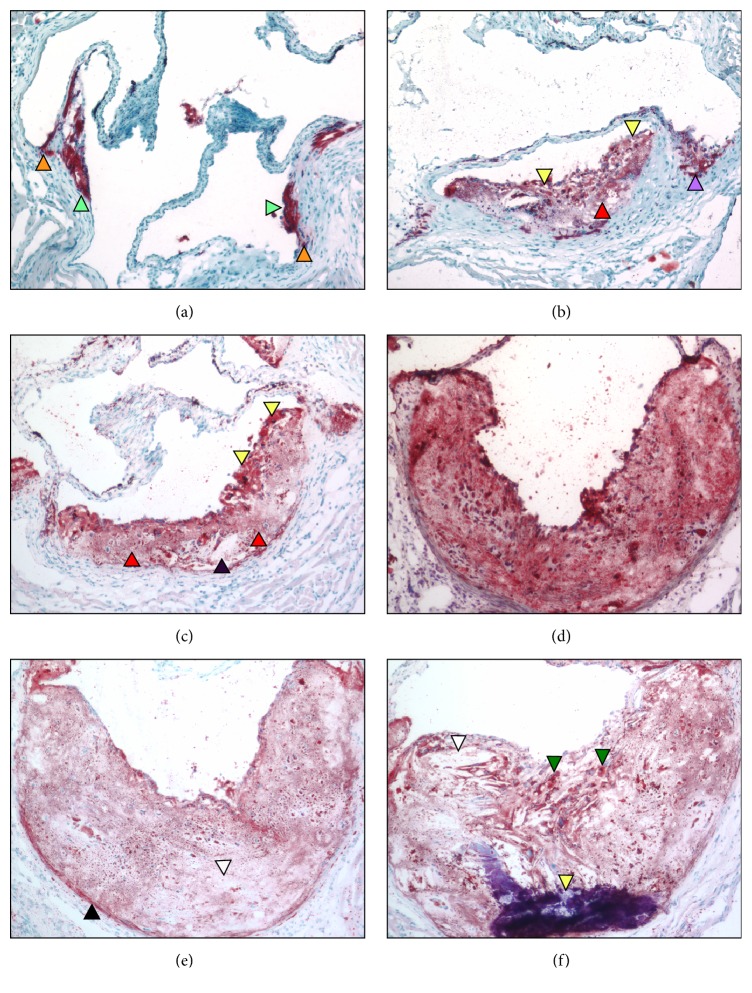
Oil Red-O stains of aortic sinuses from typical* L*^−/−^*/A*^−/−^ mouse at various ages. (a) 12 weeks of age. Small, dense, fat droplets are observed near the lumen of the sinus (green arrowheads) with minor accumulation in the media (orange arrowheads). (b) 18 weeks of age: dense lipid droplets are now seen associated with the cellular cap (yellow arrowheads) and at the base of a smaller developing plaque adjacent to the base of the valve (purple arrowhead). Light, patchy droplets are also accumulated below the cap into the focally acellular core (red arrowhead). (c) 24 weeks of age. Lipid in the cap region (yellow arrowheads), along with diffuse fat droplets (red arrowheads) and cholesterol clefts (black arrowhead) within the core. (d) 48 weeks of age. Diffuse lipid in the core and cap. (e) 60 weeks of age. The expansive lesion consists of a more complex core with diminished presence of lipid. Several areas within the core appear lipid-free (white arrowhead) but some dense accumulation is observed within the media of the thin stretched wall (black arrowhead). (f) 72 weeks of age. A more extensive cholesterol cleft formation is seen within the complex lesion extending from the base of the core to the thinned cap (white arrowhead). Multiple lipid-containing foci are observed in the subcapsular region (green arrowheads) and a large calcified region extends from the arterial wall into the core (yellow arrowhead). The surrounding plaque contains patchy, moderately sized fat droplets. Original magnification, 100X.

Oil Red-O stains of sectioned aortic sinuses from* L*^−/−^/*A*^−/−^ mice at various ages are presented in Figure 5. At 12 weeks of age (Figure 5(a)) the presence of small dense fat droplets near the lumen of the sinus with minor accumulation in the media is observed, while at 18 weeks of age (Figure 5(b)), dense lipid droplets are observed associated with the cellular cap and at the base of a smaller developing plaque adjacent to the base of the valve. Light, patchy droplets are also accumulated below the cap into the focally acellular core. At 24 weeks (Figure 5(c)), lipid is accumulated throughout the cap and into the core, with the greatest accumulation in the cap region. Large patchy areas of diffuse droplets are observed below the cap into the core and cholesterol clefts are seen within the core. At 48 weeks (Figure 5(d)), lipid is densely scattered throughout the core and cap of the growing plaque and at 60 weeks of age (Figure 5(e)) the more expansive lesion consists of a complex core with diminished presence of lipid. Several areas within the core appear lipid-free, but some dense accumulation is observed within the media of the thin stretched wall. At 72 weeks of age (Figure 5(f)), a more extensive cholesterol cleft formation is noted within the complex lesion extending from the base of the core to the thinned cap. Multiple lipid-containing foci are observed underlying the endothelium and a large calcified region is present in the base of the plaque. The surrounding plaque contains patchy, moderately sized fat droplets.

**Figure 6:**
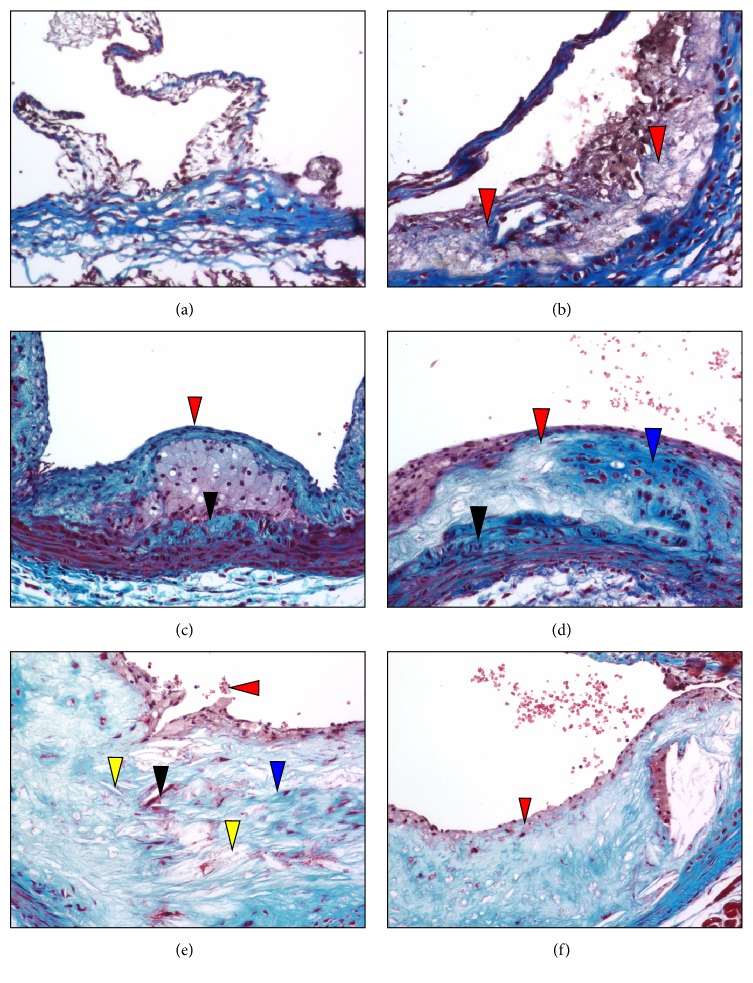
Trichrome stains of aortic sinuses from typical* L*^−/−^*/A*^−/−^ mouse at various ages. (a) 12 weeks of age. Trichrome staining indicated that the fatty deposits present in the small intimal compartment adjacent or attached to the base of the aortic valve are devoid of collagen. Original magnification, 200X. (b) 18 weeks of age. Trichrome staining shows diffuse collagen deposits (red arrowheads) between the intimal and medial compartments. Original magnification, 200X. (c) 24 weeks of age. Trichrome stains demonstrate that most of the collagen is now associated within the subcapsular region (red arrowhead) and large aggregates of collagen deposits are evident within the core (blue arrowhead) and in the arterial wall (black arrowhead). Original magnification, 100X. (d) 36 weeks of age. Trichrome stains demonstrate that most of the collagen is now associated with the subcapsular region (red arrowhead) and large aggregates of collagen deposits are evident within the core (blue arrowhead) and in the arterial wall (black arrowhead). Original magnification, 100X. (e) 48 weeks of age. Trichrome staining indicates sloughing of the lightly diffuse collagen cellular cap (red arrowhead). Patchy diffuse collagen aggregates are evident within the core (blue arrowhead). A few red-staining foci are noted in the core (black arrowhead). An increased abundance of cholesterol clefts is observed in the core (yellow arrowheads). Original magnification, 100X. (f) 60 weeks of age. Trichrome staining reveals diffusely interwoven collagen deposits in the subcapsular region (red arrowhead), with diffuse deposition in the core (blue staining). Original magnification, 100X.

Trichrome staining of sectioned aortic sinuses from* L*^−/−^/*A*^−/−^ mice at various ages are presented in Figure 6. Trichrome staining of 12-week mice indicates that they are devoid of collagen (Figure 6(a)), whereas at 18 weeks trichrome staining (Figure 6(b)) shows diffuse collagen deposits in the intimal compartment. At 24 weeks of age, Trichrome staining (Figure 6(c)) shows that the fibrous cap is collagen-rich and encapsulates a collagen negative foam cell core. Focal areas of collagen deposition are evident in the arterial wall. Focal cells in the core and a thickened arterial wall are also evident. At 36 weeks of age, Trichrome stains (Figure 6(d)) demonstrate that most of the collagen is associated with the underlying endothelial region and collagen deposits are evident within the core and in the arterial wall. At 48 weeks of age, Trichrome staining (Figure 6(e)) indicates sloughing of the lightly diffuse collagen cellular cap and patchy diffuse collagen aggregates are evident within the core. A few positive-staining foci are noted in the core, along with an increased presence of cholesterol clefts. At 60 weeks of age, Trichrome staining (Figure 6(f)) reveals diffusely interwoven collagen deposits in the underlying endothelial region, with diffuse deposition in the core.

**Figure 7:**
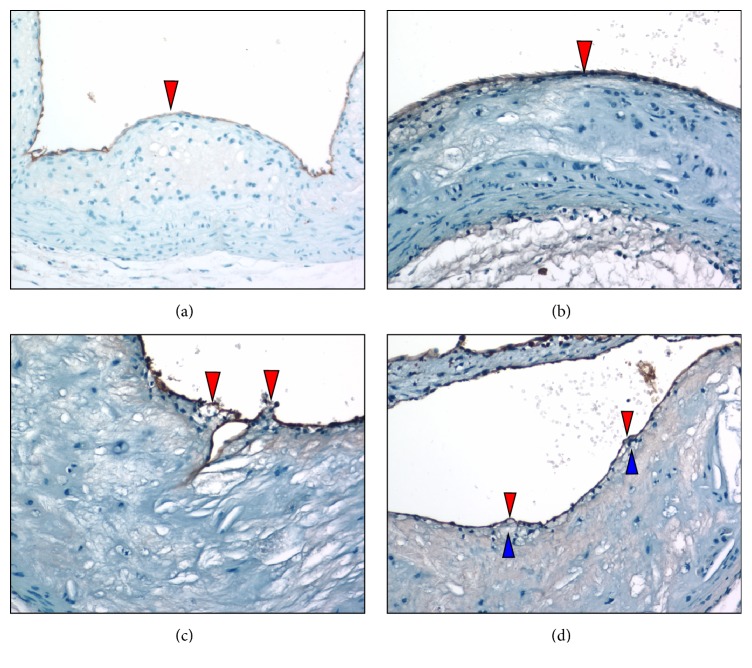
Anti-CD31 immunostaining of aortic sinuses from typical* L*^−/−^*/A*^−/−^ mouse at various ages. (a) 24 weeks of age. CD31 immunostaining indicates that an intact endothelium (red arrowhead) separates the lumen from the fibrous cap. (b) 36 weeks of age. Anti-CD31 immunostains reveal that an intact endothelium (red arrowhead) separates the lumen from the lesion. (c) 48 weeks of age. Anti-CD31 immunostaining reveals multiple focal breakpoints within the endothelial layer (red arrowheads). (d) 60 weeks of age. Anti-CD31 immunostaining indicates the presence of focal rounded areas in the endothelium, with small breakpoints (red arrowheads). Weakening of the endothelium may be the result of underlying foam cells (blue arrowheads). Original magnification, 100X.

Anti-CD31 immunostains of sectioned aortic sinuses from* L*^−/−^/*A*^−/−^ mice at various ages are presented in Figure 7. At 24 weeks of age, anti-CD31 immunostaining (Figure 7(a)) indicates that an intact endothelium separates the lumen from the fibrous cap. At 36 weeks of age, anti-CD31 immunostains (Figure 7(b)) reveal an intact endothelium separating the lumen from the lesion. At 48 weeks of age, anti-CD31 immunostaining (Figure 7(c)) reveals multiple focal breakpoints within the endothelial layer. At 60 weeks of age, anti-CD31 immunostaining (Figure 7(d)) indicates the presence of focal rounded areas in the endothelium, with a small breakpoint. There are bulging endothelial regions with underlying foam cells.

**Figure 8:**
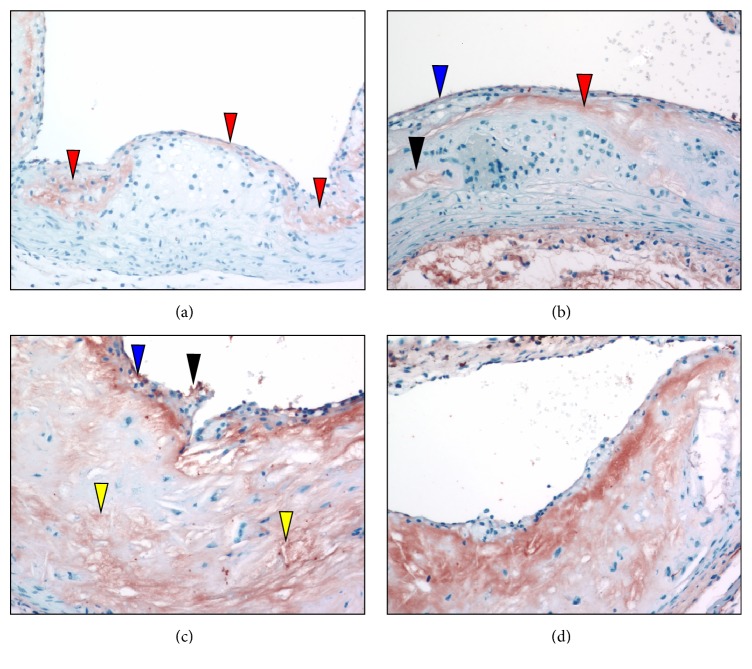
Anti-Fg immunostaining of aortic sinuses from typical* L*^−/−^*/A*^−/−^ mouse at various ages. (a) 24 weeks of age. Fg immunostains demonstrate that diffuse fibrin deposits (red arrowheads) are present in the subluminal spaces and at the base of the lesion above the media. (b) 36 weeks of age. Anti-Fg immunostains demonstrate variable deposits of fibrin within the subcapsular region (red arrowhead), as well as some focal, but faint, positive areas associated with the endothelium (blue arrowhead) and lipid core above the media (black arrowheads). (c) 48 weeks of age. Anti-Fg immunostaining demonstrates that the underlying subcapsular region is abundant in fibrin deposits (red staining) and patchy areas of fibrin are associated with the endothelium (blue arrowhead). Fibrin is also identified at breakpoints on the endothelium (black arrowhead). Diffuse areas of fibrin are evident at the base of the lipid core above the media (yellow arrowheads). (d) 60 weeks of age. Anti-Fg immunostaining demonstrates fibrin deposition (red staining) scattered throughout the subcapsular and core regions. Original magnification, 100X.

Antifibrin(ogen) immunostaining of sectioned aortic sinuses from* L*^−/−^/*A*^−/−^ mice at various ages are presented in Figure 8. At 24 weeks of age, antifibrinogen immunostains (Figure 8(a)) indicate that diffuse fibrin deposits are present in the underlying endothelial spaces and at the base of the lesion above the media. At 36 weeks of age, antifibrinogen immunostains (Figure 8(b)) demonstrate variable fibrin(ogen) in the underlying endothelial region, as well as some focal, but faint, positive areas associated with the endothelium, and in the lipid core above the media. At 48 weeks of age, antifibrin(ogen) immunostaining (Figure 8(c)) demonstrates that the underlying endothelial region is abundant in fibrin deposits. Patchy areas of fibrin are also associated with the endothelium and fibrin is also identified at breakpoints on the endothelium. Diffuse areas of fibrin are evident at the base of the lipid core above the media. At 60 weeks of age, antifibrin(ogen) immunostaining (Figure 8(d)) demonstrates fibrin deposition scattered throughout the underlying endothelium and core region.

**Figure 9:**
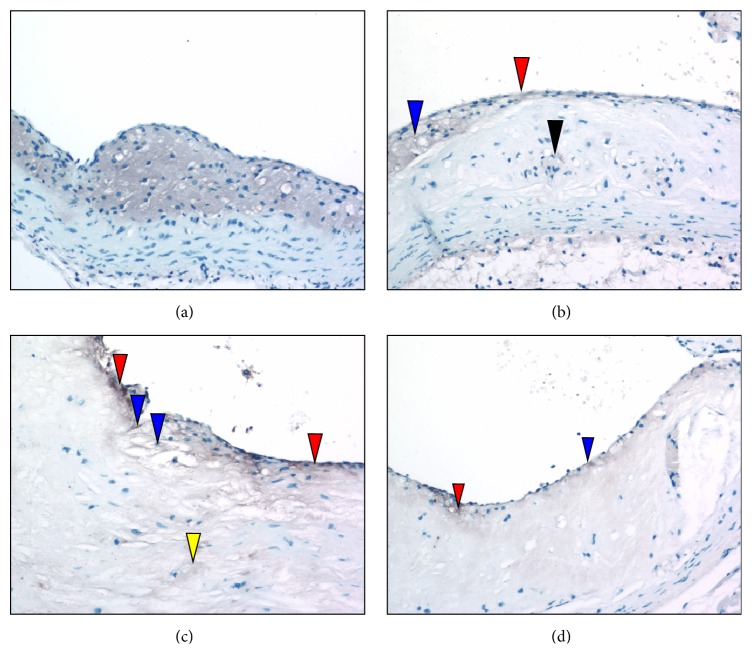
Antimacrophage immunostaining of aortic sinuses from typical* L*^−/−^*/A*^−/−^ mouse at various ages. (a) 24 weeks of age. Antimacrophage stains demonstrate that the foam cell core (gray area) consists predominantly of macrophages. (b) 36 weeks of age. Antimacrophage immunostains show that the cap now consists of scattered macrophages (red arrowhead). At the broadest area of the cap, the macrophages appear as foam cells adjacent to the core (blue arrowhead). There are a few lightly stained (gray) positive stains within the core (black arrowhead). (c) 48 weeks of age. Antimacrophage immunostaining shows macrophages (gray stains) within the thinned cap in the subendothelium (red arrowheads). Some are associated with breakpoints of the endothelium underlying some faintly positive foam cells (blue arrowheads). The central core shows focal but scattered areas of faintly stained cells (yellow arrowhead). (d) 60 weeks of age. Antimacrophage immunostaining reveals small clusters of macrophages within the endothelial (blue arrowhead) and subendothelial areas (red arrowhead). Original magnification, 100X.

Antimacrophage immunostaining of sectioned aortic sinuses from* L*^−/−^/*A*^−/−^ mice at various ages is presented in Figure 9. At 24 weeks of age, the antimacrophage immunostains (Figure 9(a)) demonstrate that the core consists predominantly of macrophages. At 36 weeks of age, antimacrophage immunostains (Figure 9(b)) show that the cap now consists of scattered macrophages. At the broadest area of the cap, the macrophages appear as foam cells adjacent to the core. At 48 weeks of age, antimacrophage immunostaining (Figure 9(c)) shows macrophages within the thinned cap in the subendothelium. Some are associated with breakpoints of the endothelium underlying some faintly positive foam cells. At 60 weeks of age, antimacrophage immunostaining (Figure 9(d)) reveals small clusters of macrophages in the endothelial and subendothelial areas.

**Figure 10:**
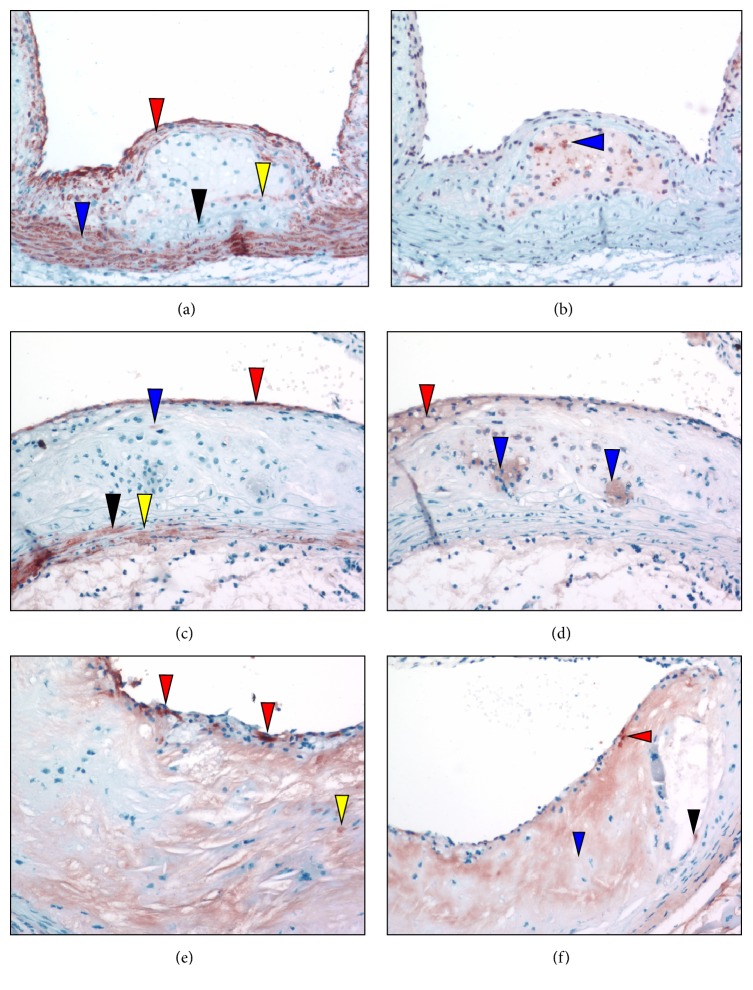
Anti-SMA and anti-SMemb immunostaining positive foci of aortic sinuses from typical* L*^−/−^*/A*^−/−^ mouse at various ages. (a) 24 weeks of age. Anti-SMA immunostaining reveals a cellular multilayered SMC *α*-actin positive region associated with the fibrous cap (red arrowhead), as well as the normally positive medial compartment (blue arrowhead). However, at the base of the lipid core, within the media, an area devoid of positive cells is evident (black arrowhead) with a faintly positive region immediately underlying the core (yellow arrowhead). (b) 24 weeks of age. Several SMemb-positive foci are identified in the core (blue arrowhead), in an area devoid of SMA positive cells. (c) 36 weeks of age. Anti-SMA immunostains indicate numerous single layered positive cells (red stain) at the endothelium. They consist of weak to highly positive SMA foci among other negative cells. Only one weakly positive cell is located under the subcapsular region (blue arrowhead). The medial compartments of the arterial wall are populated with both SMA positive (black arrowhead) and negative (yellow arrowhead) cells. The inner medial compartment (below the lipid core) appears distended and is mainly negative for SMA. (d) 36 weeks of age. Several diffuse SMemb-positive foci are identified within the broad end of the foam cell-containing cap (red arrowhead). Additional foam cell clusters within the core are also positive (blue arrowheads). These areas within the core were negative for SMA. (e) 48 weeks of age. Anti-SMA immunostains disclose SMC-actin positive (red) cells in the subluminal region, which are intensely positive (red arrowheads) and diffusely scattered among SMA negative border cells. A few positive cells are identified within the core (yellow arrowhead). (f) 60 weeks of age. Anti-SMA immunostains show SMA positive cells that are faintly visible in the subendothelium (red arrowhead) with a mostly acellular necrotic core containing few positive cells (blue arrowhead). A positive cell is identified at the base of the fragmented lesion within the core (black arrowhead). Original magnification, 100X.

Anti-SMA immunostaining of sectioned aortic sinuses from* L*^−/−^/*A*^−/−^ mice at various ages are presented in Figure 10. At 24 weeks of age, anti-SMA immunostaining (Figure 10(a)) reveals a cellular multilayered SMC *α*-actin-positive region associated with the fibrous cap, as well as with the positive medial compartment. However, at the base of the lipid core, within the media, an area devoid of positive cells is evident, with a faintly positive region immediately underlying the core. Furthermore, several SMemb-positive cells are identified in the core associated with an area devoid of SMA positive cells (Figure 10(b)). At 36 weeks of age, anti-SMA immunostains (Figure 10(c)) indicate numerous single layered positive cells at the endothelium. They consist of weak to highly positive SMA foci among other negative cells. The medial compartments of the arterial wall are populated with both SMA positive and negative cells. The inner medial compartment (below the lipid core) appears thickened and is mainly negative for SMA. Furthermore, several diffuse SMemb-positive foci are identified within the broad end of the foam cell-containing cap (Figure 10(d)). Additional foam cell clusters within the core are also positive. These areas within the core were negative for SMA. At 48 weeks of age, the anti-SMA immunostained pattern (Figure 10(e)) discloses SMC *α*-actin positive cells in the subendothelial region, which are intensely positive and diffusely scattered among SMA negative border cells. At 60 weeks of age, anti-SMA immunostains (Figure 10(f)) show SMA positive cells that are faintly visible in the subendothelium with a mostly acellular necrotic core containing few positive cells. A positive cell is identified at the base of the fragmented lesion within the core.

**Figure 11:**
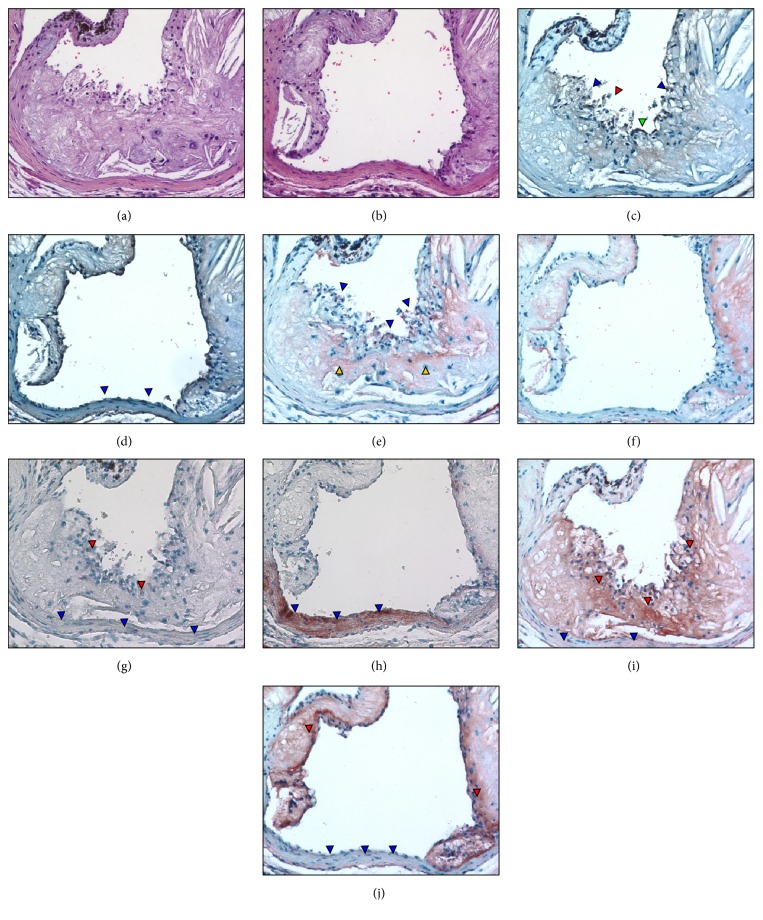
Plaque characteristics in a representative 72-week-old* L*^−/−^*/A*^−/−^ mouse at 2 different depths, one slice containing an endothelium completely covered by a plaque and the other containing an intact endothelium. (a, b) H&E stains, showing the general morphology of the aortic sinus. (c) Endothelial discontinuity is demonstrated as a disruption in endothelial cell staining on the most luminal surface of the plaque (blue arrowheads). Fragmented debris from the plaque is also observed in the lumen of the sinus (red arrowhead). An intact area of endothelium is also noted (green arrowhead). (d) Several cells on the surface of the aortic wall are stained positive for endothelial cells (blue arrowheads). (e) Dense fibrin deposits are observed on the cap (blue arrowheads), which colocalize with ruptured areas identified from endothelial cell staining. Diffuse fibrin is also present in the remaining part of the plaque core (yellow arrowheads). (f) Continued presence of fibrin in the plaque body. (g) Anti-SMA immunostains (red coloration) in a plaque area illustrating a small number of cells are positively stained (red arrowheads) in the cap area. No positive stains are observed in the aortic wall (blue arrowheads). (h) Intense anti-SMA-staining in the aortic wall (blue arrowheads) is observed in an endothelium not covered by plaque. (i) Anti-SMemb immunostaining in a plaque area showing cap (red arrowheads) and aortic wall (blue arrowheads) features. (j) Anti-SMemb immunostains are negative in aortic wall areas not covered by plaques (blue arrowheads). Original magnification, 100X.

The plaque of a representative 72-week-old* L*^−/−^/*A*^−/−^ mouse is shown in Figure 11. H&E stains at two different depths (Figures 11(a) and 11(b)) show sections covered (Figure 11(a)) and noncovered (Figure 11(b)) by plaque. The plaque contains an irregular cap with many broken areas (Figure 11(c)). Diffuse fibrin is also present in the remaining part of the plaque core (Figures 11(e) and 11(f)) and dense fibrin deposits are observed on the cap, which colocalize with ruptured areas identified from endothelial cell staining (Figure 11(e)). A small number of SMA positive cells are observed in the cap area covered by plaque (Figure 11(g)), but the aortic wall is heavily stained with SMA positive SMC cells at a depth not covered by plaque (Figure 11(h)). SMemb-positive cells were detected in the cap area of the plaque and light staining is also in the aortic walls (Figure 11(i)). No expression of SMemb is observed in aortic wall areas in sections at a depth where plaque does not cover the wall (Figure 11(j)).

## 4. Discussion

Atherosclerosis in humans begins focally in lesion-prone vascular areas where blood flow is compromised [18]. This reduced flow then facilitates recruitment of monocytes to intimal locations, via their interaction with cell adhesion molecules (CAMs), followed by monocyte differentiation to macrophages, which subsequently become foam cells. This disease then progresses in a series of AHA-defined classifications, from simple Type I to advanced Type VI lesions [19]. Complications of atherosclerosis include thinning and rupture of unstable plaques and aneurysm. Rupture of the plaque leads to thrombotic disease and possibly sudden death. Certain characteristics of plaques, including the size and composition of the lipid core, the structure and composition of the fibrous cap, apoptosis and/or dedifferentiation of collagen-synthesizing SMCs, and/or the presence of a local inflammatory process, predispose the plaque to disruption [20, 21]. Plaque rupture is frequently observed in calcified plaques [22].

The nature and level of plasma lipoproteins, especially of HDL-C, are of predictive significance in the risk of coronary artery [23] and plasma HDL-C has been found to be a potent inverse risk factor in a variety of clinical end-points of this disease [23–25]. On the other hand, high LDL levels are associated with risk of cardiovascular disease [26], mainly through formation of its oxidized product(s) [27]. oxLDL in the vessel wall originates from mild oxidation of vascular LDL [28] and circulating oxLDL stimulates vascular monocyte/macrophage infiltration [29], as well as vascular EC [30] and SMC [31, 32] migration and proliferation. Thus, it has become clear that a variety of genes, including those that influence lipid and carbohydrate metabolism, those that influence the hemodynamic state of the organism, those that mediate many phases of the inflammatory response, and those that affect hemostasis, can affect the development and progression of atherosclerosis [33], and murine models of atherosclerosis are extensively employed to investigate the effects of gene alterations on the characteristics of this disease.

In this investigation, we have employed a mouse strain that contains a total combined deficiency of the* Apobec1* and* Ldlr* genes in order to both eliminate the liver production of apoB-48 and inhibit clearance of the produced apoB-100-containing particle. This has led to a lipid profile in these mice, which is very similar to that of atherosclerotic prone humans with familial hypercholesteremia, wherein the very high levels of cholesterol reside in LDL particles. These excessive LDL-C levels then predispose these mice, even on low fat, low cholesterol diets, to spontaneous development of atherosclerostic lesions, similar to the human condition. Whereas this mouse model has been introduced in a previous study [13], a systematic characterization of the lesions that develop has not been offered.

The lesions that spontaneously develop in *L*^−/−^/*A*^−/−^ mice progressively worsen with time and begin as fatty streaks in the proximal aortic regions as early as 12 weeks of age. These lesions spread to distal regions and, by 72 weeks of age, occupy >60% of the entire arterial tree. Intimal thickening occurs with foam cells and collagen-producing SMCs, which then progresses to a fibrous cap containing atheroma, overlaying a well-established necrotic core. The fibrous cap then thins and the core becomes calcified. We have only sporadically observed mice that experience sudden death, despite clear evidence that the cap progressively thins, loses collagen-producing SMCs, perhaps through dedifferentiation, and gains macrophages, which may degrade the stabilizing collagen within the cap. This may indicate that cap rupture is not a feature of this model, a usual situation for murine models of atherosclerosis, but evidence for cap erosion is present in this model. WHHL (Watanabe Heritable Hyperlipidemic) and WHHLMI (WHHL Myocardial Infarction) rabbits are used as arteriosclerosis model animals. The function of LDLr is impaired in these rabbits due to spontaneously occurring mutations in* Ldlr*. Moreover,* Apobec1 *is not expressed in rabbit liver; thus, the majority of cholesterol is packaged in LDL fractions similar to humans [34]. However, a small number of cap ruptures are observed in these rabbit models. Cap rupture and/or cap erosion are asymptomatic without thrombotic complications even in humans. While these animals exhibit cap rupture and cap erosion, it is very hard technically to detect sudden death due to thrombotic complications. Previously, we reported that prothrombin times and activated partial thromboplastin times were shorter in *L*^−/−^/*A*^−/−^ mice than Wt mice. Moreover, the platelets and von Willebrand factors in *L*^−/−^/*A*^−/−^ mice were more activated [15]. However, considering the average lifespan of *L*^−/−^/*A*^−/−^ mice is almost equal to that of Wt mice, these mice might not expire due to thrombotic complications following cap rupture and/or cap erosion, and the severity of thrombotic complications in humans might be much worse than that in other animals.

## 5. Conclusions

In conclusion, the* L*^−/−^/*A*^−/−^ model is very suitable for the study of features of LDL-C driven spontaneous atherosclerosis in humans. It offers many advantages over the* Apoe*^−/−^ and* L*^−/−^ models in terms of generation of the initial stages of the disease and also appears to incorporate many of the progressive features of the human disease, beginning with fatty streaks, developing to a clear human stage IV atheroma and then to a thin cap fibrous atheroma. While this model is ideal for investigation of genetic influences of a variety of pathways on atherosclerosis development and progression, generation of mice with further deficiencies is a more complex and time-consuming process. Despite this, we have successfully developed desirable strains containing additional gene deficiencies. In our previous report, some of the serum parameters other than factors related to coagulation and fibrinolysis in* L*^−/−^/*A*^−/−^ mice were reported [15]. Global assessment of this model other than functions of any specific genes integrated also will be the subject of future communications.

## Figures and Tables

**Table 1:** Lipid profiles of Wt, *L*^−/−^, *A*^−/−^, and *L*^−/−^/*A*^−/−^ mice.

	*L* ^−/−^/*A*^−/−^	*L* ^−/−^/*A*^−/−^	Wt	*L* ^−/−^	*A* ^−/−^	*L* ^−/−^/*A*^−/−^	*L* ^−/−^/*A*^−/−^	*L* ^−/−^/*A*^−/−^	*L* ^−/−^/*A*^−/−^	*L* ^−/−^/*A*^−/−^
Age (weeks)	12	18	24	24	24	24	36	48	60	72

N^a^	14	16	17	14	11	45	38	26	13	17

Body weight (g)	26.3 ± 1.6	27.4 ± 2.1	28.7 ± 2.7	28.4 ± 2.4	31.7 ± 3.0	29.0 ± 2.4	29.3 ± 3.0	32.8 ± 3.2	34.5 ± 4.1	31.8 ± 2.3

Total-C^b^ (mg/dL)	341 ± 68	347 ± 66	66.6 ± 8.3	255.1 ± 45.5	74.6 ± 13.2	350 ± 79	337 ± 96	348 ± 64	335 ± 30	325 ± 77

VLDL-C^c^ (mg/dL)	22.8 ± 10.8	23.3 ± 5.5	NA	20.0 ± 7.4	NA	36.1 ± 10.3	21.4 ± 7.8	29.6 ± 11.3	21.1 ± 9.2	19.8 ± 9.8

LDL-C^d^ (mg/dL)	275 ± 60	268 ± 57	NA	163.2 ± 19.9	NA	264 ± 50	267 ± 84	274 ± 51	271 ± 23	271 ± 67

HDL-C^e^ (mg/dL)	44.1 ± 8.0	49.4 ± 9.1	NA	97.0 ± 26.1	NA	47.4 ± 16.1	43.8 ± 13.4	40.2 ± 12.2	36.2 ± 4.9	33.5 ± 11.6

LDL-C/HDL-C	6.4 ± 1.7	5.5 ± 0.7	NA	1.8 ± 0.6	NA	6.2 ± 2.6	6.3 ± 1.9	7.2 ± 1.7	7.3 ± 1.4	8.5 ± 2.0

Triglyceride (mg/dL)	168 ± 33	155 ± 22	63.4 ± 20.3	102.6 ± 28.8	75.4 ± 21.6	154 ± 28	153 ± 33	159 ± 25	157 ± 28	120 ± 22

^a^Number of mice used at each age.

^b^Total cholesterol.

^c^Cholesterol contained in VLDL.

^d^Cholesterol contained in LDL.

^e^Cholesterol contained in HDL.

## Data Availability

The data used to support the findings of this study are available from the corresponding author upon request.
